# EquiPrEP: An implementation science protocol for promoting equitable access and uptake of long-acting injectable HIV pre-exposure prophylaxis (LAI-PrEP)

**DOI:** 10.1371/journal.pone.0291657

**Published:** 2023-09-19

**Authors:** Christina M. Kaul, Brandi E. Moore, Emma Kaplan-Lewis, Eunice Casey, Robert A. Pitts, Patricia Pagan Pirallo, Sahnah Lim, Farzana Kapadia, Gabriel M. Cohen, Maria Khan, Ofole Mgbako

**Affiliations:** 1 Office of Ambulatory Care and Population Health, HIV Services, NYC Health + Hospitals, New York, New York, United States of America; 2 Division of Infectious Diseases and Immunology, Department of Medicine, NYU Grossman School of Medicine, New York, New York, United States of America; 3 Department of Epidemiology, New York University School of Global Public Health, New York, New York, United States of America; 4 NYC Health + Hospitals/Bellevue, New York, New York, United States of America; 5 Department of Population Health, NYU Grossman School of Medicine, New York, New York, United States of America; 6 NYU Langone Institute for Excellence in Health Equity, NYU Grossman School of Medicine, New York, New York, United States of America; Beth Israel Deaconess Medical Center/Harvard Medical School, UNITED STATES

## Abstract

**Background:**

Long-acting injectable HIV pre-exposure prophylaxis (LAI-PrEP) was approved by the U.S. Food and Drug Administration in December 2021. This initial phase of implementation represents a prime opportunity to ensure equitable LAI-PrEP provision to communities often underrepresented in PrEP care before disparities in access and uptake emerge. Herein, we describe the EquiPrEP Project which utilizes an equity-oriented implementation science framework to optimize LAI-PrEP rollout in an urban safety-net clinic in New York City.

**Methods:**

The primary objectives of this project are to: (1) increase LAI-PrEP initiation overall; (2) increase uptake among groups disproportionately impacted by the HIV epidemic; (3) preserve high PrEP retention while expanding use; and (4) identify barriers and facilitators to LAI-PrEP use. EquiPrEP will enroll 210 PrEP-eligible participants into LAI-PrEP care with planned follow-up for one year. We will recruit from the following priority populations: Black and/or Latine men who have sex with men, Black and/or Latine cisgender women, and transgender women and nonbinary individuals. To evaluate implementation of LAI-PrEP, we will utilize equity-focused iterations of the Reach, Effectiveness, Adoption, Implementation, Maintenance (RE-AIM) framework and the Consolidated Framework for Implementation Research (CFIR), in addition to longitudinal surveys and qualitative interviews.

**Discussion:**

Novel LAI-PrEP formulations carry tremendous potential to revolutionize the field of HIV prevention. Implementation strategies rooted in equity are needed to ensure that marginalized populations have access to LAI-PrEP and to address the structural factors that hinder initiation and retention in care.

## Background

### Persistent disparities in HIV prevention care

Deep inequalities in prescriptions of pre-exposure prophylaxis (PrEP) for HIV prevention exist across racial, ethnic, gender and sexual minority groups in the United States (US) [1–3]. Those who are most represented in HIV prevention PrEP care are not from populations currently experiencing the highest rates of incident HIV infection. For example, in 2021 approximately 42% of new HIV diagnoses were among Black/African Americans, however they only represented 14% of PrEP users [4]. Disproportionately low PrEP uptake has been demonstrated amongst other underserved populations, including Latine cisgender men and women and transgender women [1, 2, 5–9]. A recent study revealed that only 32% of HIV-seronegative transgender women reported using PrEP, even though 92% were aware of the medication [10]. Additionally, Black and Latine people have the lowest rates of oral PrEP use among all racial or ethnic groups as only 9% of Black and 16% of Latine individuals who are PrEP-eligible actually received an oral PrEP prescription in 2020 [11]. Oral PrEP usage in cisgender women is also low, with approximately 10% of PrEP-eligible cisgender women receiving an oral PrEP prescription in 2020 [11]. Importantly, structural and institutional barriers—such as inadequate insurance coverage and a dearth of providers competent in the care of racially, sexually, and gender diverse people—have contributed to the exclusion of these groups in PrEP care and HIV prevention. Ultimately, without a focus on equity in the rollout and implementation of oral PrEP programs, immense barriers were created that have contributed to disparities in PrEP awareness, PrEP discussions with health providers, and PrEP prescriptions [2, 3, 6, 8].

In December 2021, the first long-acting injectable pre-exposure prophylaxis (LAI-PrEP), cabotegravir, was approved by the U.S. Food and Drug Administration (FDA) based on the results from the HIV Prevention Trial Network (HPTN) 083 and HPTN 084 trials which demonstrated LAI-PrEP to be superior to an oral PrEP formulation (emtricitabine/tenofovir disoproxil fumarate) in preventing HIV infection among cisgender men and transgender women who have sex with men, and cisgender women, respectively [12, 13]. Given the recent approval of LAI-PrEP, little data have been published regarding optimal implementation in clinical settings. Additionally, there are anecdotal reports of challenges in implementing LAI-PrEP due to the increased frequency of visits required and the need for an interdisciplinary team approach [14]. Engaging diverse patient populations for LAI-PrEP implementation is of upmost importance given persistent structural and systems-level failures in ensuring access to HIV prevention tools for individuals who are marginalized due to their race, gender, and/or sexual identities [5–7, 11, 15, 16].

### Community and clinical collaborations to achieve equity

Since the early days of the HIV epidemic, community-engaged research has increased our understanding of health disparities and potential strategies for enhancing health equity [17–19]. Upfront involvement of the most impacted communities is considered essential in the performance of culturally competent research, specifically when targeting marginalized populations [19, 20]. However, in the current literature, there are few evaluations of the effectiveness of community-clinic partnerships in the implementation of HIV prevention delivery models, and there are no evaluations of the effectiveness of collaborations pertaining to the roll-out of LAI-PrEP [5, 15, 20, 21]. To address this gap, the EquiPrEP Project seeks to be intentional about a community-engaged approach for LAI-PrEP implementation with the goal of facilitating equitable, low barrier and culturally appropriate care access. Our team initiated the project with a series of foundational conversations with community leaders, service organizations, and other key stakeholders to inform study design and establish partnerships with community-based organizations (CBOs) to expand LAI-PrEP awareness and access.

The nature of these foundational conversations can be divided into three categories: (1) long-standing participation in community planning bodies; (2) meetings with clinical and pharmacy teams; and (3) targeted conversations with CBOs. The New York City Health + Hospitals (NYC H+H) Office of HIV Services (HIVS) has been an active partner in planning bodies related to ending the HIV epidemic in New York state, supporting HIV prevention efforts in New York State and City, and overseeing Ryan White HIV Treatment funding. These planning bodies bring together community members, clinical and service providers, and department of health staff. For the EquiPrEP Project, they provided the underlying networks used to identify resource needs, best practices, and first-person understanding from communities most affected by HIV. With the advent of long-acting injectables (LAI), the HIVS team also initiated a series of meetings with clinical and pharmacy leaders in the NYC H+H system to identify obstacles and develop resolutions for providing LAI services to patients within the NYC public healthcare system. This developed into a LAI Work Group at NYC H+H which addressed issues that included access for uninsured patients as well as any collaborations needed to support patients in care. Finally, the core of community collaboration efforts for EquiPrEP were targeted conversations with CBOs. An overview of the EquiPrEP study was provided to community forums such as existing community advisory boards in the NYC H+H research network and to CBOs with a history of supporting populations who have been underserved by current PrEP programs. Ultimately, these diverse avenues of communication between clinical and community-based entities drove aspects of the inceptive design of the EquiPrEP Program, such as the need for formalized CBO partnerships, streamlined referral processes, and addressing social needs that may serve as barriers to LAI-PrEP initiation and persistence.

### An equity-based implementation science approach

Implementation science frameworks are critical tools for guiding and evaluating the implementation of health interventions and programming. Two widely used frameworks are the Reach, Effectiveness, Adoption, Implementation, and Maintenance (RE-AIM) framework and the Consolidated Framework for Implementation Research (CFIR) [22, 23]. Both frameworks have been used in prior studies to evaluate PrEP implementation in a variety of settings and include both individual-level and settings-level elements that influence an intervention’s uptake and impact [16, 24–26]. Additionally, more expansive iterations of RE-AIM have been proposed, such as that in Shelton et al., that explicitly consider issues of *equity* and *sustainability* across the various dimensions of the framework and propose potential measures for evaluation [27]. Given the centering of health equity in the EquiPrEP Program, such iterations have been fundamental to informing the design and approach of the project’s participant recruitment strategy, data collection, community partnerships, and clinical processes to LAI-PrEP rollout.

Here, we present a protocol wherein we describe our research methods, community-engaged approach, and clinical implementation for an equitable LAI-PrEP demonstration project in selected priority populations, along with the overarching process of developing this protocol.

## Study design and methods

### Clinical setting: NYC Health + Hospitals and the Bellevue Pride Center

NYC H+H is the largest public hospital system in the United States and includes 11 acute care facilities and numerous community-based clinics. Bellevue Hospital is one of the major referral centers in NYC H+H and the oldest public hospital in the country. As the city’s safety-net hospital system, NYC H+H provides care for all New Yorkers regardless of sexual orientation, gender identity, ability to pay, or immigration status. Additionally, over the last decade, NYC H+H has established seven Pride Centers across the system designed to address healthcare needs specific to the lesbian, gay, bisexual, transgender and queer (LGBTQ) communities [28].

The EquiPrEP Program was conceptualized by the NYC H+H Office of HIV Services (HIVS) and HIV clinical leadership across NYC H+H. HIVS is the coordinating office for HIV prevention and care, supporting the 11 New York State Department of Health Designated AIDS Centers and six HIV community clinics that provide HIV primary care to over 12,000 New Yorkers. HIVS is comprised of a clinical director for quality care who is an infectious disease specialist, senior director, clinical informatics support and data analysts, program and grants managers, and a community/patient liaison.

The Bellevue Pride Center was established in June 2018. Staffing includes four primary care medicine providers, one psychiatrist, one social worker/therapist, two coordinators, and one administrative supervisor. The Bellevue Pride Center is embedded within Ambulatory Clinical Care Services, which enables all Bellevue Pride patients to have access to additional resources, including case management, financial counseling, and subspecialty consultation. To date, the Bellevue Pride Center is responsible for the care of over 400 patients, the majority of whom self-identify as African American/Black or Black Hispanic (19%), Latine or White Hispanic (22%), or White (22%). Approximately one-fifth of all patients are Spanish-speakers and 14% are uninsured—illustrative of the underserved and diverse patient population the Pride Center supports. In addition to comprehensive gender-affirming care, the Pride Center also specializes in HIV prevention and sexual health, including screening and treatment of sexually transmitted infections (STIs). Further, the Pride Center has strong internal partnerships with the Bellevue Emergency Department and OB/GYN clinic—sites with historically high STI testing—to ensure all PrEP-eligible patients who access Bellevue services are successfully linked to longitudinal HIV preventive care [29].

### Community-based partners

The EquiPrEP Project sought to develop long-term relationships with specific CBOs based on organizations’ experiences successfully advocating for, and providing services to, populations that have been underserved by HIV prevention programming. The guiding principles of our partnerships were rooted in tenets of “centering in the margins,” a concept which posits that focusing on the needs of those most often marginalized in HIV prevention and bringing them to the forefront of our efforts will challenge inherent power differentials in HIV research and provide the foundation for our health equity approach [30]. Prior to designing the study, research team members held several meetings with relevant stakeholders involved in HIV prevention services across NYC—such as the New York State Department of Health AIDS Institute—to identify CBOs that serve priority populations of racial, gender and sexual minorities and that had interest in providing HIV prevention services. From this, four CBOs were identified for study partnership: Destination Tomorrow, African Services Committee, Alliance for Positive Change, and Latino Commission on AIDS. These CBOs were subsequently contacted and a series of meetings were planned to introduce the EquiPrEP Project and possible partnership.

To establish a foundation for these meetings and related discussions, we developed a brief but comprehensive presentation that covered the following: (1) relevant background on HIV PrEP disparities; (2) Bellevue Pride Center history and current resources available; (3) preliminary specific aims and protocol design for EquiPrEP; (4) priority populations of interest for enrollment and eligibility criteria; (5) potential referral processes; and (6) potential benefits of collaboration and strategies for resource-sharing if partnerships were established. These meetings were run by the project’s principal investigators and a patient navigator dedicated to the EquiPrEP Project. Leaders of CBOs with decision-making capacity attended as well as CBO staff with significant client interactions such as client navigators, case managers, HIV testing personnel, and program coordinators.

During meetings, CBOs were encouraged to provide feedback and voice concerns regarding both our protocol design and current PrEP delivery strategies. Such conversations frequently informed important changes to the proposed study protocol and clinical activities. For example, Destination Tomorrow—a CBO largely dedicated to the support of transgender persons—quickly identified problematic aspects of LAI-PrEP for many transgender women. Buttocks fillers and implants were excluded from clinical trials of LAI-PrEP and because they are commonly used in gender-affirming care, many transgender persons may not be able to access or benefit from LAI-PrEP [31]. Given that transgender women represent one of the most vulnerable populations for HIV infection, the EquiPrEP team discussed the need for additional work to study the safety of alternative body site administration and initiated active conversations with pharmaceutical companies to explore potential solutions. Additionally, the Latino Commission on AIDS voiced concerns about their clients who are undocumented and/or uninsured being able to participate in the study and receive medical care. In order to agree to a partnership with the EquiPrEP Project, the organization wanted further details on how this subset of clients would be targeted for enrollment and supported in accessing LAI-PrEP. This prompted the study team to incorporate immigration status as a dimension for assessing study reach and for informing recruitment strategies. Additionally, clinical staff were trained on options for care coverage for undocumented and/or uninsured individuals and the process of enrolling such individuals into care. Representatives from all CBOs also raised understandable concerns about the sustainability of LAI-PrEP care after study completion. In response to this, the EquiPrEP team reviewed proposed clinical workflows to ensure that the systems and resources established for the study could be feasibly integrated into standard clinical care after the study concludes to prevent interruptions in care.

To enhance buy-in after preliminary meetings, CBOs were informed that individuals referred to EquiPrEP would be assessed for all services provided at our safety net hospital, including gender affirming primary care, mental health care, and subspecialty consultation. The EquiPrEP team also proposed to compile lists of services available at partnered CBOs and to train the EquiPrEP patient navigator to refer already established Bellevue patients and EquiPrEP participants to these CBOs as indicated. Ultimately, through longitudinal bi-directional relationships with CBO partners, the EquiPrEP Project aims to facilitate the sharing of resources that enhance the care provided to patients while simultaneously reaching interested persons from populations who have had limited access to LAI-PrEP.

As expected and welcomed, when partnerships were being formalized, CBOs offered significant feedback on referral processes and the nature of partnerships. For example, the Latino Commission on AIDS recommended referrals be made in real-time via video calls as this method was most helpful for clients, would allow for the secure exchange of client information, and would enhance the likelihood of successful linkage to EquiPrEP. After implementing CBOs feedback, the EquiPrEP team developed unique memoranda of understanding (MOU) for each CBO that outlined bi-directional referral processes, listed the resources shared between organizations, and solidified our partnerships. These MOUs were agreed upon and signed by leaders of the respective organizations, though they are considered live documents that can be revisited and revised as needed. Additionally, all CBOs requested regularly scheduled check-ins to establish a channel of communication for open feedback and recruitment updates. Beyond these scheduled meetings, representatives from CBOs have visited the Bellevue Pride Center and our associated events for patients. Similarly, EquiPrEP team members have attended events hosted by CBOs to table, speak, and provide information about EquiPrEP to community members. Participation in and support of such events was essential for demonstrating our sincere intent to establish genuine partnerships with CBOs.

Overall, the utilization of a community-engaged research approach and the development of a strong bi-directional relationship with CBOs is vital to the success of the EquiPrEP Project. These collaborations will help foster recruitment from and retention of our priority populations, allowing us to efficiently connect participants to trusted and respected community-based service providers and shift our implementation strategy as needed.

### Study design and sample

The EquiPrEP study design was collaboratively developed through a partnership between the NYC H+H/Bellevue Pride Center, NYC H+H/Bellevue Infectious Disease Division, New York University School of Medicine Department of Population Health, New York University School of Global Public Health, and NYC H+H Office of HIV Services. The primary objectives of the EquiPrEP Project are to: (1) increase LAI-PrEP initiation overall; (2) increase LAI-PrEP uptake among groups disproportionately impacted by the HIV epidemic; (3) to preserve high LAI-PrEP retention while expanding use; and (4) to identify barriers and facilitators to LAI-PrEP use that may be unique to urban safety-net populations including addressing social determinants of health (SDOH) and connecting patients to resources from various CBOs.

**Fig 1:**
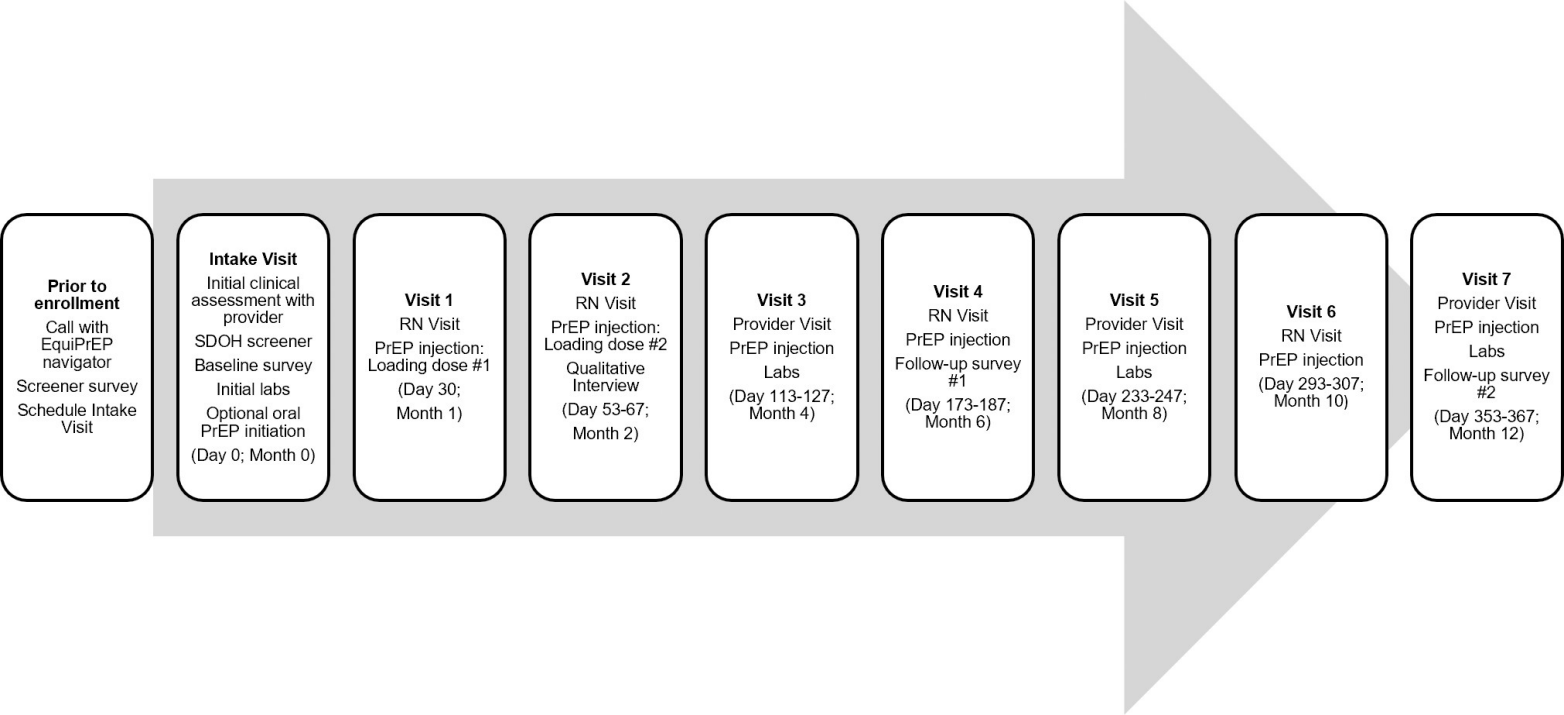
Timeline of participant visits and study activities.

To achieve these objectives, the study will involve 12 months of follow-up of an intended cohort of PrEP-eligible individuals who are interested in initiating LAI-PrEP. We propose a multi-method study design that involves the collection of both qualitative and quantitative data at key study visits during the course of the project. The proposed timeline for study activities and clinic visits is summarized in Fig 1.

In consideration of clinic capacity, we set a target sample size of n = 210 participants. The study sample will include Black and/or Latine men who have sex with men (MSM), Black and/or Latine cisgender women, and transgender women and nonbinary individuals. We plan to specifically recruit participants from these populations because they have not had equal access to or uptake of PrEP and therefore have not benefited from this vital HIV prevention tool to the same extent as other populations. Thus, we will employ a non-probability quota sampling strategy to recruit 70 individuals from each priority population. Overall, inclusion criteria will be as follows: aged 18 years or older; HIV-seronegative status; Black/Latine MSM, Black/Latine cisgender woman, transgender woman, or non-binary individual; agreeable to initiation of LAI-PrEP, reside in New York state; and fluent in English and/or Spanish. These criteria were defined based on study objectives, PrEP prescription guidelines, and study capacity. Importantly, potential participants will be eligible for study inclusion regardless of insurance status or ability to pay because patients with no insurance or inadequate coverage will be directed to Bellevue staff to facilitate enrollment in insurance and/or to enroll the patient in PrEP assistance programs.

### Participant recruitment and enrollment

Participants will be recruited both internally from the NYC H+H patient population and externally via referral from our network of partnered CBOs. More specifically, eligible patients will be recruited into the study through the following sources: (1) external referrals from community partners: referral protocols, linkage agreements and ongoing communication systems have been established with CBOs that advocate for, and provide supportive services to, study priority populations; (2) internal Bellevue referrals from priority clinics such as the Women’s Health clinic; (3) internal NYC H+H electronic referrals; (4) PrEP-eligible patient flags: NYC H+H has established an indicator within the electronic health record (EHR) that identifies individuals eligible for PrEP based on their recent STI testing and diagnostic history. This list will be reviewed to identify opportunities to outreach to the patient and offer enrollment into the study; (5) positive bacterial STI patient lists: the Pride Center maintains an active list of patients who test positive for *Neisseria gonorrhoeae* and/or *Chlamydia trachomatis* within the past seven days at Bellevue. This list will be reviewed daily to identify patients requiring treatment for bacterial STIs who may need HIV prevention services; and (6) Patients already engaged at Bellevue’s Pride Center requesting transition from oral to injectable PrEP.

All individuals that are referred to the EquiPrEP Project will be contacted by the EquiPrEP patient navigator via telephone who will assess an individual’s eligibility for study participation based on patients’ self-reported answers. After a patient is determined to be eligible for the study, an intake appointment will be scheduled where participants are provided with detailed information on the study and informed consent is acquired. An individual’s capacity to consent will be determined by the study team members consenting potential participants. Evaluation of capacity to consent is based on consideration of the following factors: participants’ ability to communicate a specific treatment choice, ability to understand the risks/benefits of a treatment choice and study participation, ability to describe clinical procedures, and ability to express a choice about whether or not to participate [32]. Once a participant is enrolled, baseline laboratory testing will also be conducted at this intake visit (including HIV antigen/antibody and viral load testing) and medication will be ordered (injectable cabotegravir with or without optional one-month oral lead in with oral cabotegravir depending on patient preference). After confirmation of HIV negative status, the EquiPrEP patient navigator will focus on the authorization process for injectable cabotegravir to ensure that the date of first injection is within one week of negative HIV testing. In instances where authorization takes longer than one week, repeat HIV antigen/antibody and viral load testing will be performed. Importantly, during the period from enrollment until first injection or oral cabotegravir lead-in initiation, all participants will be offered oral PrEP with Truvada as a bridge to prevent HIV acquisition in the waiting period before injectable cabotegravir authorization.

Enrollment will be evaluated twice monthly to ensure recruitment targets are met. Enrollment by referral source will also be evaluated periodically to increase recruitment of individuals from our priority populations if current enrollment numbers are not sufficient.

### Clinical implementation

#### Medication procurement

Prior to beginning patient enrollment, a medication procurement/prior authorization workgroup was organized including an infectious disease pharmacist, the Bellevue Hospital Pride Center providers and the H+H clinical quality director. The purpose of this group was to streamline the workflow related to the authorization process and procurement of injectable cabotegravir. As described above, the inception of this study coincided with H+H systems-level efforts to facilitate access to LAI HIV treatment and prevention medications. System-level efforts included the following: EHR decision support and patient tracking tools; discussion with pharmacy leadership to add LAI medication onto H+H systems-level formulary; educational forums open to any H+H employee regarding clinical use of LAI medications for HIV treatment or prevention as well as pharmacy and billing components of medication procurement and administration. All resources were placed within the health system intranet which can be accessed systemwide by all H+H staff and is routinely used by ambulatory care leadership to disseminate clinical decision support tools.

Facility pharmacies were also trained on the details related to injectable medication procurement and pharmacy claim adjudication. Because H+H is the municipal healthcare network in NYC, all patients are welcome regardless of ability to pay and, as such, it is the responsibility of the health system to provide any intervention/medication to any patient who comes through the door. Thus, an additional component of the medication procurement/authorization workgroup was to outline pathways to obtain LAI medication for any potential scenario including for patients who are privately insured, insured through Medicaid, and uninsured. From this, relevant staff could be trained on both medical and pharmacy benefit procurement as well as prescription assistance programs such as Viiv Connect. Overall, this groundwork helped ensure the clinic was prepared to enroll participants, procure LAI-PrEP, and determine avenues for LAI-PrEP coverage for all participants.

#### Clinical staff

A major pillar of the EquiPrEP implementation strategy is the addition of staff to support the clinicians administering LAI-PrEP. These additions include: (1) a bilingual patient navigator trained in culturally competent LGBTQ+ care, structural racism and its impact on healthcare delivery/outcomes, LAI-PrEP, and motivational interviewing. This individual will help recruit individuals for study participation, schedule and track participant appointments, and address access or retention obstacles for patients including those related to SDOH-related concerns as identified by a SDOH screener; and (2) a clinical pharmacist who will support patient education and understanding of LAI medications, steward the prior authorization process, and support the successful coordination with pharmacies dispensing LAI-PrEP and education of patients on side effects, drug-drug interactions and other medication-related issues. We believe the integration of these roles in the clinical care delivery process will be integral to successful implementation of LAI-PrEP.

#### Clinical workflow

Once a participant is enrolled in the study and has completed their intake appointment and prior authorization has been approved for LAI-PrEP, they will follow an injection and HIV/STI testing schedule that aligns with the 2021 Centers for Disease Control and Prevention Guidelines on PrEP [13]. As seen in Fig 1, participants will be seen every two months for injections and relevant labs and injection visits will alternate between physician visits and nursing visits with physician visits occurring every other injection visit (i.e., every four months).

Missed injection appointments will be followed-up with three outreach attempts by the EquiPrEP patient navigator: two attempts by phone and one by mail. For participants who miss their injection appointments but who ultimately return to the clinic, FDA label guidance will be followed such that a re-loading dose will be required if more than one month has passed since the originally scheduled injection appointment. With any missed injections that require re-loading, a bridge with either Truvada or oral cabotegravir will be offered and repeat HIV antigen/antibody and viral load testing will be confirmed to be negative prior to re-initiating injections. To reduce risk of missed injection appointments and loss to follow up, participants will receive a phone call reminder several days prior to their appointment and all participants will be given a direct contact to the study team to facilitate communication and flexibility in instances of scheduling changes.

All study participants will be tracked within a secure spreadsheet that is managed by the EquiPrEP patient navigator and accessed by Bellevue Hospital Center Pride Center providers involved in delivering LAI-PrEP. This spreadsheet will be used to determine upcoming appointment dates and track participants from enrollment to medication authorization, initial injection appointment, and subsequent injection appointments. Additionally, clinical staff will utilize EHR-based tools and shared patient list features to communicate when medication authorization came through, when medication has been delivered, and when an injection is due or overdue.

### Data collection

#### Participant surveys

All potential participants will complete a brief screener survey to assess their eligibility for study participation. Individuals who decline to enroll in the study will have the choice to complete a short exit survey that documents their reasons for declining LAI-PrEP. For those who choose to enroll in the study, a baseline survey will be administered during the initial intake visit. The baseline survey will collect data on sociodemographic characteristics including participants’ race, ethnicity, gender, sexuality, health insurance, highest education level, immigration status, employment status, housing status, and incarceration experience. The survey will also inquire about participants’ relationship and sexual history including participants’ relationship/marital status, number of recent sexual partners and partners’ genders, past history of intimate partner violence, concurrent partnerships, and history of STIs. Additional topics explored in the baseline survey include participants’ substance use history and frequency, perceived HIV risk, potential concerns with LAI-PrEP, and reasons for pursuing LAI-PrEP rather than oral PrEP. Perceived HIV risk will be measured using items from the Perceived Risk of HIV Infection Scale (PRHS) developed by Napper and colleagues [33]. To assess concerns about LAI-PrEP, participants will be asked to rate their level of worry about various aspects of LAI-PrEP (e.g., injection frequency, side effects, level of protection) from 1 (not at all concerned) to 4 (very concerned) [34]. The baseline survey will be delivered via REDCap in either English or Spanish and will take approximately 25 minutes to complete. Participants will receive a $25 gift card for survey completion.

Follow-up surveys will occur at the 6-month and 12-month visits and will be similar in scope and content to the baseline survey while also including questions about participants’ experiences with LAI-PrEP side effects, their perceptions of the general experience of receiving LAI-PrEP, and potential barriers to remaining retained in LAI-PrEP care. Measures assessing the acceptability of LAI-PrEP product attributes and the physical experience of injection will be derived from prior work by Tolley and colleagues on LAI-PrEP acceptability [35]. Potential barriers to care retention that are examined will include transportation difficulties, challenges with insurance, experience with side effects, and experiences with PrEP-related stigma as well as clinic attributes such as appointment times, visit length, and participants’ relationships with clinical staff. Additionally, if participants choose to transition from LAI-PrEP to oral PrEP or cease all PrEP use during the study period, these follow-up surveys will also collect data related to individuals’ reasons for discontinuing LAI-PrEP. Each follow-up survey will take approximately 20 minutes to complete and participants will be provided with a $15 gift card upon completion of each survey.

For participants that disenroll from the study, we will attempt to administer a brief discontinuation survey that asks about participants’ reasons for disenrolling, their HIV risk perception, experience with LAI-PrEP side effects, and potential barriers they faced while attempting to receive LAI-PrEP care.

#### Social determinants of health (SDOH) assessments

In the past, uptake of biomedical prevention tools and health services in general has been shown to be impacted by unmet social needs [5]. Further, the relationship between unmet SDOH needs and uptake and adherence to LAI-PrEP is unclear. Given these issues, the EquiPrEP Project aims to identify social needs that could potentially affect a participant’s uptake and adherence to LAI-PrEP using a SDOH screening tool that is widely used across the NYC H+H system as standard of care.

Participants will first be screened for social needs during their intake visit at the Pride Center. The brief SDOH screening tool assesses the following needs: assistance with medical costs; support to enroll in cash assistance programs; transportation assistance; housing insecurity and homelessness; food insecurity; legal services; environmental issues at home; support for utilities; education or English language learning support; and childcare. After the intake visit, participants’ SDOH needs will be re-assessed during every other injection visit and changes in indicated needs will be tracked via REDCap.

Participants screening positive for any needs will be connected to a large network of support service providers or resources within the NYC H+H system and from our CBO partners. A closed loop referral provides a real-time view of the status of a referral between the referring provider, the patient, and the referral organization. NYC H+H utilizes a closed loop referral process with community providers to match patients to convenient services and to improve communication between service providers and care team members. This referral process occurs on an online personalized community referral platform that also captures outcome data to monitor workflows and referral success including comprehensive metrics on needs identified, referrals made, referral service types, and referral outcomes. Follow-up calls with participants who indicate a need will also be conducted to assess their awareness of referrals, receipt of any services/resources following referrals, and whether the participant believes the need has been addressed or if further referrals are needed.

### In-depth qualitative interviews

A subset of 30 study participants will be recruited for individual, in-depth interviews via convenience sampling. These interviews will occur during the 2-month follow-up visit. Trained research staff will conduct these interviews in English or Spanish using a semi-structured interview guide that inquires about barriers and facilitators to LAI-PrEP uptake and adherence. Additionally, these interviews will also ask about participants’ social needs, if and how they were addressed, and how this impacted uptake and adherence to LAI-PrEP care. Participants will be provided a $50 gift card for completion of an in-depth interview.

Additionally, all members of the clinical care team and study implementation staff (n = 15) will also engage in individual in-depth interviews. These interviews will also be conducted with a semi-structured interview guide that focuses on challenges and successful implementation strategies, with particular emphasis on obtaining contextual factors at the clinical, hospital, and systems-level that serve as barriers and facilitators. Interviews with community partners will also be conducted to examine processes, functioning, and outcomes of community engagement.

All interviews will be audio-recorded, transcribed, and translated (Spanish interviews only). Interviews will last between 20 to 45 minutes and will be conducted at the Pride Center, in-person or over the phone, depending on participant preference.

### Ethical considerations

The study will be conducted in accordance with the Declaration of Helsinki, and is approved by the Institutional Review Board (or Ethics Committee) of Bio-medical Research Alliance of New York (protocol 22-12-747; approved November 4, 2022). Participants will be properly instructed and indicate that they consent to study participation by signing informed consent paperwork.

### Project evaluation

**Fig 2:**
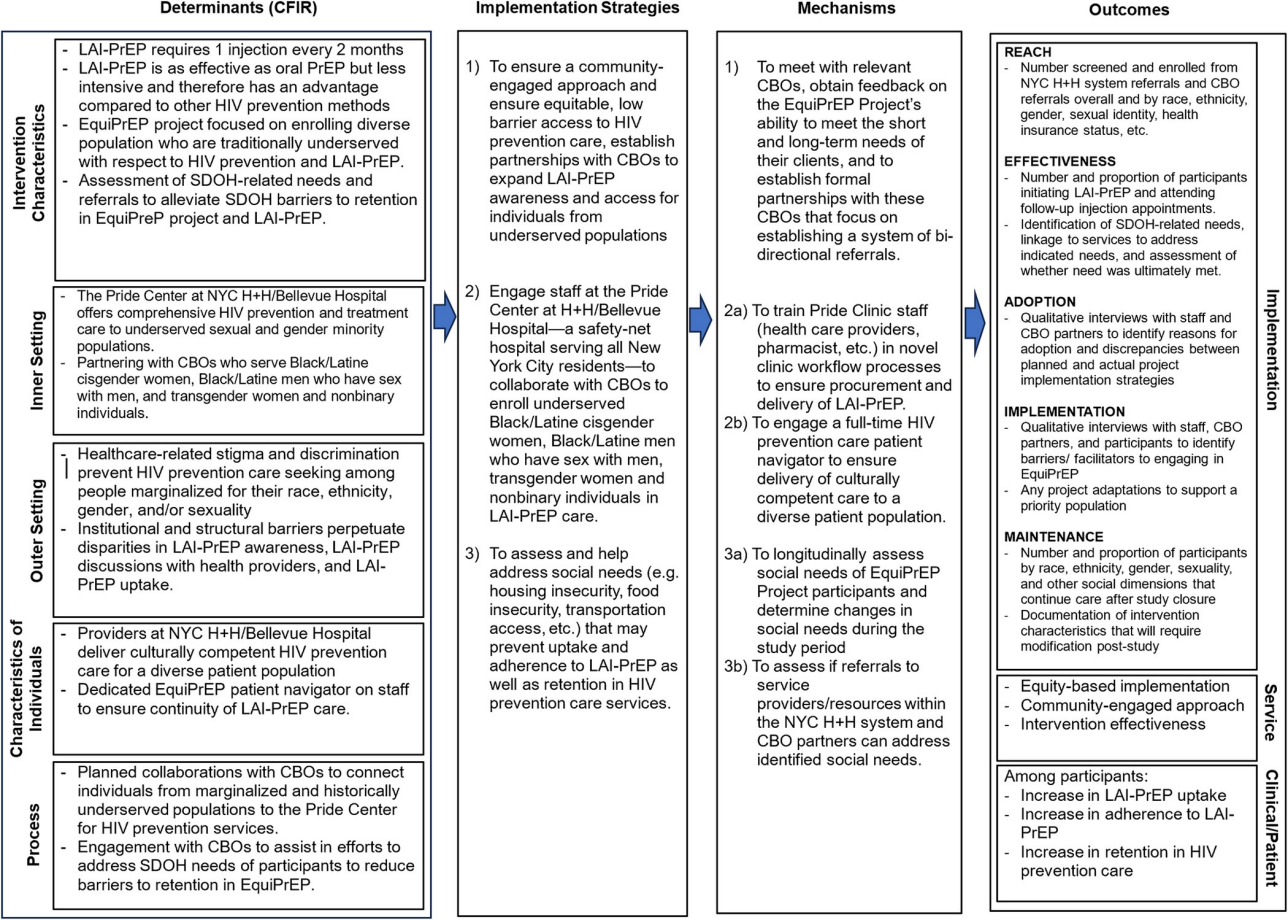
An implementation research logic model for the EquiPrEP study.

To evaluate our approach for LAI-PrEP implementation, we intend to use an equity-focused iteration of the RE-AIM framework [27]. In Shelton et al, the authors state “equity and costs are foundational driving forces across RE-AIM dimensions that shape sustained impact and warrant the need for initial and ongoing adaptation.” Interim analyses of data from participants’ surveys, EHRs, SDOH screeners, and in-depth interviews throughout the project will allow us to adapt our strategy to ensure we are actively considering issues of inequity that arise and utilizing aspects of our implementation strategy (e.g. EquiPrEP patient navigator, CBO partnerships) to address them. Additionally, as mentioned prior, we are actively attempting to screen for social needs and address social/structural determinants of health through an implementation strategy that integrates EHR-based SDOH screenings, internal and CBO-partner referrals, and follow-up in quantitative and qualitative assessments regarding how an identified domain of the SDOH (e.g., food insecurity, lack of transportation) may impact LAI-PrEP care retention and whether needs have been addressed over time. A logic model that outlines how LAI-PrEP will be implemented and evaluated in the EquiPrEP Project is depicted in Fig 2.

Our primary indicators of “Reach” will be the absolute number of individuals from the populations of interest that are screened and successfully enrolled into the study, as well as the representativeness of those who enroll in regards to factors such as race, ethnicity, gender, sexuality, health insurance status, immigration status, and referral source. Data relevant to these indicators will be captured in the screener and baseline surveys. Interim analyses using descriptive statistics will be conducted throughout the study period to determine the number and representativeness of individuals who are screened for eligibility, enrolled into the study, and successfully initiated on LAI-PrEP. Given the study’s focus on equity, we are particularly interested in identifying challenges with recruitment for our different priority populations and, more specifically, whether we are successfully reaching and enrolling individuals who may face considerable structural barriers to LAI-PrEP care such as those with incarceration experience, housing instability, or uninsured status. Brief reports on recruitment and enrollment numbers will be presented to the EquiPrEP team twice monthly and results will inform if and how recruitment strategies should be modified to address gaps in reach.

Indicators of “Effectiveness” will include the absolute number and proportion of enrolled participants who actually initiate LAI-PrEP and who are subsequently retained in LAI-PrEP care as measured by their attendance at follow-up injection appointments throughout the study period. To incorporate considerations of equity in our evaluations of effectiveness, we will also use descriptive statistics to determine whether there is differential retention among participants on the basis of various social dimensions such as race and gender. If interim analyses reveal that one participant population is experiencing a disproportionate amount of missed injection appointments, then targeted retention strategies will be implemented to enhance support for those participants. Additionally, the EquiPrEP Project’s ability to address patients’ SDOH while they receive LAI-PrEP care will also be assessed via periodic SDOH screenings and direct follow-up with participants and CBOs to determine whether participants were successfully linked to requested resources. In follow-up surveys, data will also be collected on whether various unmet needs and socio-structural determinants—such as transportation access, stigma, or housing instability—impacted participants’ ability to attend injection appointments and remain in LAI-PrEP care. Analyses of SDOH and follow-up survey data will utilize descriptive statistics to determine: (1) the number and proportion of participants who report that a SDOH-related need was addressed or is in the process of being addressed by an EquiPrEP referral; (2) the number and proportion of participants who report delaying or missing a LAI-PrEP appointment due to various unmet needs or socio-structural barriers; and (3) differences in the former outcomes by social dimensions such as race, ethnicity, gender, and sexuality. Results from such analyses will inform the extent to which processes related to referrals and resource-linkage should be altered to better support vulnerable participants.

To evaluate “Adoption”, we will utilize data from in-depth qualitative interviews with staff and community partners to identify reasons for project participation and any discrepancies between the actual implementation of the intervention and original implementation plans. Evaluations of “Implementation” will also utilize data from in-depth interviews with staff to identify barriers and facilitators to consistent intervention delivery and any adaptations that were made to the intervention over the study period. In regards to equity, any adaptations made to the intervention that were done to enhance support of participants from specific populations are of particular interest and will be identified in qualitative data and documented and acted upon by the study team. Additionally, participant perspectives on barriers and facilitators to continued program participation will also be ascertained through data from follow-up surveys and in-depth interviews. We intend to further organize and analyze all qualitative data related to intervention implementation via the menu of constructs proposed by the Consolidated Framework for Implementation Research [23].

Finally, indicators of “Maintenance” will include the absolute number and proportion of participants from the sample who intend to continue care after study closure—outcomes ascertained through participants’ responses on the final follow-up survey—as well as documentation of intervention components that will remain unchanged after study closure and those that will require modification to ensure continued care delivery. Descriptive statistics will be used to identify differences in the proportion of participants who intend to continue care within our primary priority populations—findings which will prompt further reflection on what intervention components should be institutionalized and whether further modifications are needed to better support vulnerable populations long-term.

## Conclusions

The EquiPrEP Project will allow us to learn from the past mistakes made with oral PrEP rollout that led to inequitable access and disparities in PrEP use. By intentionally centering priority populations often left behind and leveraging genuine community partnerships, we intend to develop a LAI-PrEP implementation strategy that (1) reaches populations disproportionately impacted by the HIV epidemic and (2) addresses socio-structural barriers to care retention. If successful, we hope to chart a path for equitable dissemination of and access to LAI and other clinical innovations that are often out of reach for the communities that need them most.

## List of abbreviations

PrEP: Pre-exposure Prophylaxis
LAI-PrEP: Long-acting Injectable Pre-exposure Prophylaxis
CBO: Community-based organization
RE-AIM: Reach, Effectiveness, Adoption, Implementation, Maintenance
CFIR: Consolidated Framework for Implementation
SDOH: Social Determinants of Health
